# Challenges of measuring accurate estradiol levels in aromatase inhibitor‐treated postmenopausal breast cancer patients on vaginal estrogen therapy

**DOI:** 10.1002/prp2.330

**Published:** 2017-07-05

**Authors:** Polly Niravath, Raksha Bhat, Mohamed Al‐Ameri, Ahmed AlRawi, Claudette Foreman, Meghana V. Trivedi

**Affiliations:** ^1^ Houston Methodist Cancer Center Houston Texas; ^2^ Department of Medicine Weill Cornell Medicine New York New York; ^3^ Department of Pharmacy Practice and Translational Research University of Houston College of Pharmacy Houston Texas; ^4^ Lester and Sue Smith Breast Cancer Baylor College of Medicine Houston Texas

**Keywords:** aromatase inhibitors, breast cancer, estradiol levels, vaginal estrogen

## Abstract

Breast cancer patients who are taking adjuvant Aromatase Inhibitor (AI) therapy typically have extremely low estradiol levels, which are undetectable by routine clinical laboratories. Thus, it becomes difficult to assess the safety of interventions such as low‐dose vaginal estrogen, which may increase estradiol levels. In this study, we aimed to assess the utility of enzyme‐linked immunosorbent assay (ELISA) to measure low estradiol concentrations in breast cancer survivors on AI therapy treated with either vaginal estrogen or lubricant for atrophic vaginitis as a part of clinical trial. The samples were tested using two independent ELISA kits. Some of the samples were also evaluated using liquid chromatography‐tandem mass spectrometry (LC‐MS/MS) for comparison. We found that while the results by ELISA were reproducible, they were not accurate when compared to LC‐MS/MS. It is possible that medications or supplements may cross‐react with the ELISA reagents and confound the assessment; however, those were often not the reason for the discrepancy. Our results highlight the need for developing novel, reliable, and clinically accessible assays to measure ultra‐low estradiol levels to improve care of breast cancer survivors. At this stage, based on our findings, we recommend using MS‐based assays for estradiol quantitation for breast cancer survivors, whenever necessary.

**Abbreviations**
AI: aromatase inhibitor
ER+: estrogen receptor‐positive

## Introduction

Breast cancer is the most common malignant disease in women in the Western world. In the United States, SEER data state that over 207,000 women are diagnosed with invasive breast cancer every year (Schiff et al. 2010). The majority of these women are greater than 50 years old, and approximately 80% of breast cancer cases in this age group are positive for hormone receptors (estrogen receptor, ER; progesterone receptor, PR) (Coombes et al. 2004). While tamoxifen was part of the standard of care for patients with hormone‐responsive breast cancer, its use in the adjuvant setting has largely been replaced in postmenopausal women by the third‐generation aromatase inhibitors (AIs) based on studies demonstrating superior efficacy of adjuvant AIs in early breast cancer (Dowsett et al. 1995; Howell et al. 2005; Mouridsen et al. 2009).

The third‐generation AIs include nonsteroidal aromatase inhibitors, anastrozole and letrozole, as well as the steroidal aromatase inhibitor, exemestane. These AIs suppress estrogen synthesis to nearly undetectable circulating levels by inhibiting the enzyme aromatase, thereby blocking the conversion of adrenal androgens into estrogens (Geisler et al. 1996). While this strategy is extremely effective in fighting ER+ breast cancer, AI treatment also results in vaginal dryness and sexual dysfunction for more than 60% of women (Crandall et al. 2004). The tissues in the vagina, vulva, urethra, and bladder trigone all contain estrogen receptors and thus undergo atrophy as a result of estrogen deprivation due to AI treatment. This results in decreased vaginal tissue elasticity and decreased vaginal fluid secretion, which typically leads to dyspareunia. Moreover, low estrogen levels promote higher vaginal pH (ranging from 5.5 to 6.8) which causes loss of lactobacilli and overgrowth of other bacteria, thus increasing risk of urinary tract infections (Ponzone et al. 2005; Mac Bride et al. 2010).

For non–breast cancer patients with postmenopausal atrophic vaginitis, local or systemic estrogens are very effective and are widely accepted as standard of care (Hulvat and Jeruss 2009). However, it is not known whether this approach is safe in breast cancer survivors who are taking estrogen deprivation therapy. Any potential increase in estradiol level could theoretically increase the risk of breast cancer. The studies have been conflicting as to whether exogenous estrogen therapy increases risk of breast cancer recurrence in survivors (O'Meara et al. 2001; von Schoultz and Rutqvist 2005; Cella et al. 2006). Furthermore, it is difficult to study the safety of low‐dose local/vaginal estrogen treatment in women on AI therapy because their estradiol levels are extremely low, often below 3 pg/mL (Dixon et al. 2008). However, many of the clinically available assays have a lower limit of detection of approximately 20–30 pg/mL (Dixon et al. 2008). Consequently, it is very challenging to monitor small fluctuations in estradiol levels as a measure of safety for breast cancer survivors using vaginal estradiol treatment. This severely limits our ability to conduct safe clinical trials using low‐dose vaginal estrogen therapy for the treatment of AI‐induced atrophic vaginitis. Furthermore, because we have not been able to reliably measure such low levels of estradiol, we do not know the clinical significance of mildly elevated estradiol levels in breast cancer survivors.

The gold standard for measuring estradiol remains isotope dilution gas or liquid chromatography‐tandem mass spectrometry (GC‐MS/MS or LC‐MS/MS), which may quantitate estradiol levels down to 1–3 pg/mL (Ketha et al. 2015). However, the MS‐based assays are far too complex, expensive, and time consuming for routine clinical use at this point (Rollins 2013; Rosner 2013). Recently, immunoassays for evaluating estrogen levels have shown utility in various studies (Naessen and Rodriguez‐Macias 2002; Kenemans et al. 2009). These immunoassays are thought to be cost effective and easy to conduct. The objective of this study was to determine the utility of measuring serum estradiol concentrations by enzyme‐linked immunosorbent assay (ELISA) in selected breast cancer survivors on AI therapy.

## Materials and Methods

### Patients

Estradiol concentrations were measured in eight breast cancer survivors on AI therapy treated with either vaginal estrogen Estring^®^ or nonestrogen vaginal lubricant Replens^TM^ for atrophic vaginitis as a part of clinical trial REVIVE (Replens™ Versus Vaginal Estrogen; NCT01984138). This study, conducted at the Lester and Sue Smith Breast Center, is still ongoing, but the analysis presented here examines the estradiol quantitation using ELISA for the first eight women who were enrolled on the trial. The enrolled patients were postmenopausal women with history of stage I–III Estrogen Receptor‐positive (ER+) breast cancer, who were taking adjuvant AI therapy (see Table 1). The trial randomized these women in a 1:1 fashion to receive either Replens™ or Estring^®^ (a vaginal ring which locally secretes low‐dose estrogen). All women complained of vaginal dryness and/or dyspareunia or had experienced three or more urinary tract infections in the last year. Patients were not allowed to use any exogenous estrogen within the preceding 4 weeks from study initiation. Subjects were also excluded if they were deemed to have any vaginal infections. All patients received baseline gynecologic exams and vaginal pH measurement. The study protocol was approved by the institutional Investigational Research Board, and all patients signed an informed consent.

**Table 1:** Patient demographics with history of stage I–III estrogen receptor‐positive breast cancer taking adjuvant aromatase inhibitor therapy

Patient#	Age (years)	Aromatase inhibitor	Treatment for vaginal atrophy
1	46	Letrozole	Estring^®^
2	61	Letrozole	Replens^™^
3	51	Letrozole	Replens^™^
4	53	Exemestane	Replens^™^
5	58	Letrozole	Estring^®^
6	57	Letrozole	Replens^™^
7	62	Exemestane	Estring^®^
8	62	Anastrozole	Replens^™^

Patients randomized to Replens™ were instructed to apply the cream intravaginally three times each week during the study period, whereas the women randomized to receive Estring^®^ were initially instructed on ring placement at their first gynecology visit on study. At that visit, the first ring was also placed by the gynecologist. Women had the option of placing the second ring on their own, or having the gynecologist place the second ring. The ring was replaced every 3 months, for the total study duration of 6 months.

### Estradiol measurement using ELISA assay

Blood was collected at baseline for all study patients treated with either Estring^®^ or Replens™. For those receiving Estring^®^, additional blood samples were also collected at weeks 2, 4, 8, 12, 16, and 20 to follow serum estradiol levels. Those patients randomized to Replens™ only had estradiol quantitation at baseline and at end of study. Serum was isolated and was transported on ice to UH College of Pharmacy laboratory, where it was aliquoted immediately and stored in −80°C. To measure estradiol concentrations, two different ELISA kits (Catalog# KAQ0621, Invitrogen and Catalog # IB78239, Immuno‐biological Laboratories) were used per manufacturers' protocols. Briefly, for the Invitrogen kit, 50 *μ*L of each of the samples or standards was added into the wells, followed by 50 *μ*L of estradiol‐HRP (estradiol conjugate to horseradish peroxidase) and 50 *μ*L of antiestradiol antibodies. The plate was incubated for 2 h at room temperature on a horizontal shaker set at 700 rpm. The liquid was removed and the wells were washed with wash buffer four times. Next, 200 *μ*L of chromogen solution (tetramethylbenzidine) was added, and the plate was incubated in the dark for 30 min at room temperature. The reaction was stopped with the addition of 50 *μ*L of stop solution (1.8 N H_2_SO_4_). The solution color changed from blue to yellow and absorbance was read at 450 nm. For the IBL kit, 100 *μ*L of each of the samples or standards was added into the precoated wells with antiestradiol antibodies, followed by 200 *μ*L of estradiol‐HRP (estradiol conjugate to horseradish peroxidase) and adequate mixing. The plate was incubated for 2 h at room temperature on a horizontal shaker set at 700 rpm. The liquid was removed and the wells were washed with wash buffer three times. Next, 200 *μ*L of chromogen solution (tetramethylbenzidine) was added, and the plate was incubated for 30 min at room temperature. The reaction was stopped with the addition of 100 *μ*L of stop solution (1.8 N H_2_SO_4_). The solution color changed from blue to yellow, and absorbance was read at 450 nm within 10 min of stopping the reaction. For both kits, a standard estradiol curve using a nonlinear regression analysis using the four‐parameter logistic equation performed with Graphpad Prism^®^ (version 5.0c) was used to compare the absorbance values and to derive pg/mL estradiol concentration.

### LC‐MS/MS

Some of the samples were also sent to the Clinical Laboratory Improvement Amendments (CLIA)‐certified Associated Regional University Pathologist laboratories (ARUP; Salt Lake City, Utah) for analysis by LC‐MS/MS as validated and described before (Kushnir et al. 2008). Briefly, the aliquots of 200 *μ*L of internal standards and patient serum were extracted with 1.2 mL of methyl t‐butyl ether, transferred into a 96 well plate, and evaporated under nitrogen at 50°C. These dried residues were redissolved in 50 *μ*l of dansyl chloride (3.7 mmol.L) in a 1:1 mix of acetonitrile and aqueous sodium carbonate (10 mmol/L) followed by incubated the plate in a heating block at 60°C for 10 min. A mixture of 50 *μ*l of acetonitrile and water (1:1) was added to each sample which was then analyzed by LC‐MS/MS (Kushnir et al. 2008).

## Results

### Estradiol levels by ELISA in breast cancer patients on AI

**Figure 1:**
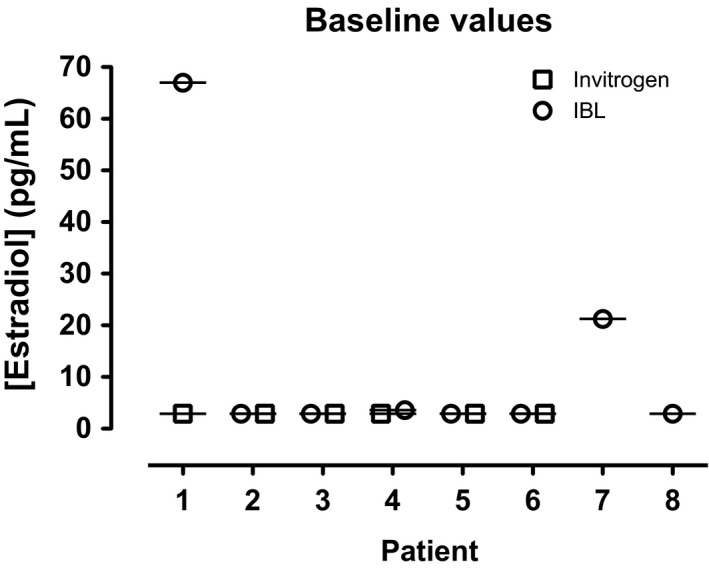
Measurement of estradiol levels in breast cancer survivors on AI therapy by ELISA. Estradiol levels were assessed in serum samples of eight breast cancer survivors on adjuvant AI by ELISA with kits from two different manufacturers, Invitrogen and IBL. All eight samples were examined by IBL kit; six of the eight samples assessed using the Invitrogen kit. Six of eight values by IBL kit were below detection limit of 3 pg/mL. Concordance between both kits was found for five of six patients. Deviation was noted for only patient #1, where estradiol value was less than 3 pg/mL by Invitrogen kit, whereas it was 67 pg/mL by IBL kit.

Estradiol values were measured by ELISA for eight breast cancer patients on an AI randomized to either Estring^®^ or Replens™ (Table 1). All eight samples were assessed by the IBL kit; whereas six of these eight samples were also assessed using the Invitrogen kit. All the values determined by the Invitrogen kit were below the detection limit of 3 pg/mL. On the other hand, six of eight values by IBL kit were below the detection limit of 3 pg/mL (Fig. 1). Concordance between the Invitrogen and IBL kit was found for five of six patients. For one patient, the estradiol value was <3 pg/mL by Invitrogen kit; whereas it was 67 pg/mL by IBL kit.

### Estradiol levels in breast cancer patients on AI being treated with vaginal estradiol over time by ELISA in comparison with LC‐MS/MS analysis

**Figure 2:**
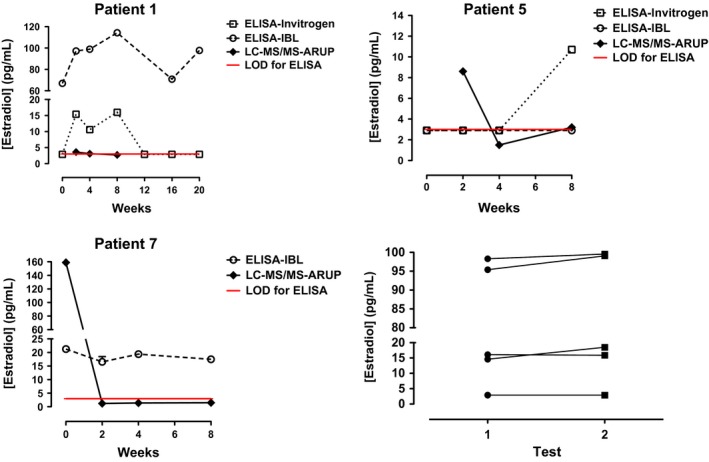
Comparison of estradiol levels in breast cancer patients on AI being treated with vaginal estradiol by ELISA versus estimation by LC‐MS/MS method: The serum samples were collected at various intervals from three patients who were on Estring^®^ to measure estradiol concentrations by two different ELISA kits, Invitrogen, and IBL. A few of these samples were also submitted for LC‐MS/MS analysis conducted by ARUP laboratories for the comparison purposes. The red line indicates limit of detection (LOD) for ELISA. 2A. Patient #1 was on letrozole and it was found that estradiol levels were varied between two different ELISA kits. 2B. The estrogen levels measured for patient #5 showed similar results by two ELISA kits at baseline, week 2 and week 4 (<3.0 pg/dl); whereas for week 8, the levels by Invitrogen kit was higher than IBL kit. Discrepancy was also found in week 2 results (8.6 pg/mL) by LC‐MS/MS analysis. 2C. The baseline estradiol results for patient #7 who was taking exemestane were highest at baseline determined by both IBL kit and LC‐MS/MS assay. 2D. Retesting of a few stored samples was done and consistency in the estradiol values was observed.

For three patients (#1, #5, and #7) on Estring^®^, serum samples were collected at baseline and at various intervals to measure estradiol concentrations by two different ELISA kits. Some of these samples were also submitted for LC‐MS/MS analysis for comparison. For patient #1, estradiol levels measured during therapy with Estring^®^ were different between the two ELISA kits at various time points (Fig. 2A). While the estradiol concentration measured by LC‐MS/MS in the samples collected at weeks 2, 4, and 8 were low (range: 2.7–3.6 pg/mL), the concentration measured by Invitrogen kit was in medium range (10.6–16.1 pg/mL) and by IBL kit was in high range (95.4–114.3 pg/mL) (Fig. 2A). This patient was taking letrozole for her AI therapy, which excluded potential interactions that have been reported between immunoassay and a steroidal AI such as exemestane (Mandic et al. 2017).

For patient #5 on letrozole, Invitrogen and IBL kits yielded similar results for baseline, week 2, and week 4 (all <3.0 pg/dl) (Fig. 2B). Whereas for week 8, the estradiol level by the Invitrogen kit was higher than that by the IBL kit (10.7 vs. <3 pg/mL). The samples submitted for LC‐MS/MS analysis showed discrepancy in only week 2 results (8.6 pg/mL), which were higher compared to both ELISA. The estradiol levels by LC‐MS/MS analysis for weeks 4 and 8 were 1.5 and 3.2 pg/mL, respectively (Fig. 2B), these values were consistent with the ELISA results.

Patient #7 was taking exemestane as her AI therapy (Table 1). Her estradiol concentrations were measured using only the IBL ELISA kit. The estradiol values ranged from 14.6 to 21.3 pg/mL during baseline to week 8, with the highest value obtained at baseline (Fig. 2C). Similarly, LC‐MS/MS assay obtained high levels at baseline (159.0 pg/mL) compared to weeks 2–8 (1.2–1.5 pg/mL).

To determine if the source of variation in the data obtained by the two ELISA kits, we retested some of the aliquoted and stored samples we had already tested. The consistency in the values of the estradiol was maintained upon retesting (Fig. 2D), suggesting the source of variation to be the inherent differences in the assay.

## Discussion

The estradiol values using different testing methods varied considerably among different ELISA platforms and LC‐MS/MS throughout our study. For example, Patient #1 had some of the most significant variations in estradiol level. Both of the ELISA assays (Invitrogen and IBL) were not only disparate from each other, but also the LC‐MS/MS results. Patient #5, on the other hand, a higher estradiol levels at week 2 by LC‐MS/MS compared to ELISA. Assuming that the baseline values were lower than that, this may reflect a brief initial surge after starting vaginal estrogen therapy. This increase in estradiol level typically resolves later, presumably as the vaginal epithelium recornifies (Kendall et al. 2006). Additionally, both of the ELISA assays were unable to capture this small increase at week 2. Unexpectedly, the Invitrogen kit measured an elevated estradiol level of 10.7 pg/mL at week 8 while her level by LC‐MS/MS was only 3.2 pg/mL. The reason for this discrepancy remains unclear. For both patients #1 and #5, there were no medications or supplements that could be identified as potential confounders. On the other hand, patient #7 was taking exemestane for her AI therapy. We suspect that this may have falsely elevated her estradiol level on the IBL kit. It is known that exemestane can interact with the ELISA reagents on some kits, resulting in a spuriously high estradiol value (Mandic et al. 2017). Thus, her estradiol levels may have varied from 14.6 to 21.3 because of a cross‐reaction with exemestane. However, it remains unclear why the baseline sample measured 159 pg/mL on the LC‐MS/MS assay. We suspect that this may have been a laboratory error as the LC‐MS/MS assay revealed estradiol values <2 pg/mL for weeks 2, 4, 8, and end of study.

Our results illustrate the difficulty in measuring ultra‐low estradiol levels in postmenopausal women on AI therapy. Although MS‐based assays such as LC‐MS/MS are considered to be the gold standard for sensitive estradiol quantitation, they are not widely clinically available due to its expense and impracticality in the clinical setting (Rosner et al. 2013). The more widely available ELISA assays have not been proven to be sensitive or reliable enough to reasonably dictate care based on our results. This remains a significant limitation in treating women with ER+ breast cancer. In trying to treat some of these women with low‐dose estrogen, we cannot adequately assess the safety of this approach if we cannot reliably measure the serum estradiol. Similarly, with the advent of ovarian suppression in premenopausal women from the SOFT and TEXT trial results (Pagani et al. 2014; Francis et al. 2015), it is becoming more important to accurately measure estradiol levels in this population too, who receive GnRH agonists injections as well as AI therapy to reduce estrogen production.

Below we discuss some of the strengths and limitations of this study. First, we found that estradiol values at baseline by ELISA match with what is reported in the literature for postmenopausal women with breast cancer on AI therapy (Kendall et al. 2006). However, these results need to be replicated in larger sample size with side‐by‐side comparison of the ELISA assay with MS‐based assays. In addition, exemestane is known to potentially interact with ELISA antibodies. We anticipated that the women on exemestane (patients #4 and #7) may have falsely elevated estradiol levels. Patient #4 had a slightly higher estradiol level than expected when measures on the IBL kit only (3.6 pg/mL). More noticeably, estradiol levels for patient #7 measured consistently higher than expected on the IBL kit at baseline, as well as weeks 2, 4, and 8, ranging from 14.6 to 21.2 pg/mL. This suggests that the IBL kit may have had more cross‐reactivity with exemestane than the Invitrogen kit. The future validation studies should focus initially on the women who are on nonsteroidal AI. If the results by ELISA or any other methods are consistent with LC‐MS/MS, further interrogation should include spiking various concentrations of exemestane in control serum samples to determine the possibility and nature of interaction. Additionally, it is possible that other endogenous hormones and exogenous medications can also cross‐react with the antibodies from the ELISA assay, which could be one reason for the wide variability seen in several patients' estradiol levels. Finally, some of the samples that were sent for LC‐MS/MS testing were more than 6 months old, which calls the quality of the sample into question. The instructions for the assay recommend that the samples are not older than 1 month if frozen; however, the stability data for older samples have not been established. The future studies should be conducted in relatively fresh samples to compare various assays. Despite these limitations, our study is an attempt to evaluate other platforms that can serve in lieu of LC‐MS/MS approach and to assess estradiol concentrations in breast cancer patients on AI therapy while also receiving vaginal estrogen.

Without accurate, ultra‐sensitive estradiol quantitation, it is extremely challenging to treat vaginal dryness in breast cancer survivors (Sulaica et al. 2016). It may also become difficult to gauge whether premenopausal women have achieved adequate ovarian suppression with GnRH agonists. Thus, we call for a more concerted effort from the scientific community to improve and standardize measurement of extremely low estradiol levels. In the same way that estrogen receptor staining has been standardized, and is widely available at most centers, we recommend that ultra‐sensitive estradiol quantitation should also be clinically available and routinely utilized for breast cancer patients. This would also allow us to answer many clinical question moving forward – Are lower estrogen levels associated with improved breast cancer recurrence rates? Is complete or near‐complete estrogen suppression necessary in premenopausal women to achieve superior results compared to tamoxifen? Do overweight women have significantly higher estrogen levels than normal‐weight women? Does this difference correlate with a clinically significant difference in outcome?

While we await better tools to answer these questions, we can only speculate. We postulate that our patients deserve better. In treating atrophic vaginitis for women on AI therapy, we need to better understand the risks and benefits of small fluctuations in the serum estradiol level.

## Disclosure

The authors declare that they have no conflict of interest.
